# Percutaneous computed tomography-guided core biopsy for the diagnosis of mediastinal masses

**DOI:** 10.4103/1817-1737.37948

**Published:** 2008

**Authors:** Suyash Kulkarni, Aniruddha Kulkarni, Diptiman Roy, Meenakshi H. Thakur

**Affiliations:** *Department of Radiodiagnosis, Tata Memorial Hospital, Mumbai, India*

**Keywords:** Biopsy, masses, mediastinal

## Abstract

**AIM:**

To describe various approaches of computed tomography (CT)-guided core biopsy and evaluate its ability to obtain adequate tissue for the assessment of mediastinal masses.

**MATERIALS AND METHODS:**

Between February 2004 and October 2006, 83 percutaneous CT-guided biopsies of mediastinal lesions were performed on 82 patients under local anesthesia. Coaxial needles were used and minimum of 3-4 cores were obtained. Post-biopsy CT scan was performed and patients observed for any complications. Tissue samples were taken to Pathology Department in formalin solution.

**RESULTS:**

From the 83 biopsies, adequate tissue for histological diagnosis was obtained in 80 (96%), and the biopsy was considered diagnostic. Of the 80 diagnostic biopsies, 74 biopsy samples were definitive for neoplastic pathology and 6 biopsy samples revealed no evidence of malignancy. There were no major complications. Minor complications were recorded in 5 patients.

**CONCLUSION:**

Percutaneous image-guided core biopsy of mediastinal lesions is an accurate, safe and cost-effective tool for the initial assessment of patients with mediastinal masses.

## Introduction

Image-guided percutaneous needle biopsy is a reliable technique for the diagnosis of thoracic lesions, particularly for assessment of mediastinal lesions.[1–3] Percutaneous image-guided mediastinal biopsies are usually performed with computed tomographic and ultrasonographic guidance, which allows precise localization and documentation of the biopsy needle and target lesions. Computed tomography (CT) is established as the best imaging method for needle biopsy guidance.[4] CT provides detailed images and helps localization of even small lesions located in any part of mediastinum. It helps in accurate planning of the needle biopsy, avoiding inadvertent puncture of vascular structures and vital organs. Multiplanar reformations with multidimentional computed tomography give valuable information about the trajectory of the needle. Different techniques for CT-guided percutaneous needle biopsy of mediastinal lesions have been advocated.[5–8]

There are several alternative methods of obtaining tissue samples from mediastinal lesions for histopathological analysis. These include surgical techniques such as thoracoscopy, cervical mediastinoscopy and anterior mediastinotomy; and needle biopsy techniques such as transbronchial needle biopsy and endoscopic ultrasound-guided fine needle aspiration biopsy.[9–11]

Percutaneous image-guided transthoracic needle biopsy allows access to virtually all mediastinal compartments, including those that are inaccessible with other alternative techniques.

## Materials and Methods

A retrospective analysis of percutaneous CT-guided biopsies of mediastinal lesions done between February 2004 and October 2006 was performed. The data collection was done with respect to demography, size of lesion biopsied, biopsy technique used, number of attempts, histopathological diagnosis and complications if any.

### Patient population

This study included 82 patients who underwent 83 percutaneous CT-guided biopsies for the diagnosis of mediastinal lesions. Out of these, 55 were male and 27 females, of age range 1 year to 73 years [Table 1].

**Table 1:** Clinical characteristics of the patients

Total number of patients	82
Adults	74
Pediatric	08
Number of biopsies	83
Reattempt to biopsy	01
Diagnostic biopsies	80/83 (96%)
Neoplastic	74
Non-neoplastic	06
Surgical confirmation	24

### Tumor characteristics

Masses from all compartments of mediastinum were sampled and included in the study.

### Biopsy techniques

The biopsies were performed with GE light-speed multiplanner diagnostic-CT scanner. All patients referred for the procedure had previous imaging done either with CT or fusion positive emission tomography-CT.

A full coagulation profile, hemoglobin level, platelet count and serum for cross-matching were available before every biopsy. Platelet counts and international normalized ratio were routinely measured in all patients. A platelet count of at least 50,000 and INR value of less than 1.5 was prerequisite for the procedure. Patients with history of, or laboratory values suggesting, abnormal coagulation were admitted before the procedure. The coagulation profile was corrected before taking the patient for procedure.

Procedures were performed under local anesthesia, deep sedation with local anesthesia or general anesthesia. In cooperative adult patients (74), the biopsies were performed under local anesthesia with continuous pulse oximetry and noninvasive blood pressure monitoring.

In pediatric patients (8), the procedure was performed under deep sedation with local anesthesia (6) or under general anesthesia (2). General anesthesia was planned when medically indicated or for patients in whom respiratory control was needed for lesion stability.

All the pediatric patients were referred to the Department of Anesthesia for pre-anesthetic checkup. Pediatric patients with an American Society of Anesthesiologists status grade II and higher were admitted before the procedure.

In view of intravenous contrast administration during the procedure, the adult patients were kept fasting for 4-6 h. For the purpose of sedation or general anesthesia, the pediatric patients were kept fasting for 6-8 h. Informed written consent for biopsy was obtained from all patients. In pediatric patients, consent for anesthesia and biopsy was obtained from parents.

In each case, previous imaging studies were reviewed to determine the best approach for percutaneous biopsy. The approach to a biopsy of mediastinal lesion in a given patient depends on multiple factors, including the clinical circumstances, the location and size of the target lesion and the presence of comorbid conditions. Patients were placed supine, prone or in lateral decubitus position, depending on the location of the lesion and safe approach for needle placement.

A diagnostic CT scan using thin axial sections was obtained after intravenous contrast administration to delineate vascular structures and differentiate vessels from lymph nodes. Then, the most suitable slice was chosen to reach the lesion and avoid injury to vascular structures and vital organs. This location was marked on skin surface with axial laser beam localizer incorporated in the CT gantry. Radiopaque markers were positioned along this laser light beam. A scan was then repeated at 2.5 mm interval to localize the exact point of entry and depth of the lesion from the skin surface.

Radiopaque markers were then removed and a mark placed on skin with an indelible pen. The depth of the lesion was calculated on the scanner in the plane of biopsy trajectory. The skin entry site was prepared in surgical fashion with savalon, povidine iodine and spirit. Local anesthetic, 5-10 ml of 1% xylocaine without adrenaline, was administered at the skin puncture site and along the expected needle tract.

A small dermotomy was made at the entry site with no. 11 blade. The coaxial needle was introduced to a predetermined depth as judged on the scan. A repeat localized CT scan was performed for the area of interest to confirm the needle position into the target lesion and for avoidance of vital structures. If inadequate, the needle position was readjusted. Trucut needle assembled in automated firing gun was then advanced through the coaxial needle and core biopsy taken.

The biopsies were performed using coaxial trucut biopsy system (Cook's quick core). The needles used were 18 G/20 G of 9 cm/15 cm/18 cm length with 10 mm/15 mm/20 mm throw. The needle selection depended on depth of lesion from skin surface, size of lesion to be biopsied and proximity to vital structures. Smaller needle diameter (20 G) was used in high-risk patients. Patients were considered to be at high risk if they had a history of coagulopathy, an American Society of Anesthesiologists status of >III or a lesion near a vital structure.

Coaxial technique helps to avoid number of passes, reducing the risk of inadvertent injury to vital structures and reducing the time of procedure.

On an average, 3-4 cores were obtained in all patients. A post-biopsy CT scan was performed after the needle removal to check for complications.

Tissue samples were taken fresh to the Histopathology Department in formalin solution. Tissue samples were collected in different tubes if they were to be sent for molecular studies.

The patients were observed for 1-3 h after the procedure to ensure their hemodynamic stability and to monitor their respiratory status. The admitted patients were shifted to the wards with continuous monitoring. Expiratory chest radiographs were obtained 3 h after the biopsy in patients in whom the pleural surface had been punctured during the biopsy procedure.

### Confirmation of findings

Verification of the percutaneous biopsy results was done with surgical pathology findings, bone marrow biopsy, biochemical features, functional imaging, patients′ response to appropriate therapy, correlation with known primary tumour in cases of metastases and clinical follow-up examination for at least 6 months.

## Results

From the 83 biopsies, adequate tissue for histological diagnosis was obtained in 80 (96.4%), and the biopsy was considered diagnostic. The remaining 3 biopsy samples on histological analysis revealed fibrocollagenous, fibrous and inflammatory tissue and were considered nondiagnostic. A reattempt to biopsy was made in only 1 patient. The size of the lesions sampled ranged from 1.5 cm to 6 cm.

Out of these three nondiagnostic biopsies, one was a case of anterior mediastinal mass with signs of superior vena cava obstruction under evaluation. The CT-guided biopsy sample from this mass on histological analysis revealed fibrocollagenous tissue only. A reattempt was made to sample the mass. Histopathological analysis of second biopsy sample revealed classical Hodgkin's disease, mixed cellularity. Another case was of a posterior mediastinal mass operated outside, surgical pathology suggestive of malignant tumor. Postoperative CT scan revealed residual mass in the posterior mediastinum. CT-guided biopsy was performed from this mass, which on histopathological analysis revealed fibrous tissue only. In view of high index of clinical and imaging suspicion, the posterior mediastinal mass was excised with wedge resection of adjacent lung. Surgical pathology analysis revealed high-grade pleomorphic sarcoma. Third case was of an ill-defined anterior mediastinal mass of the pre-vascular compartment, under evaluation. CT-guided biopsy from the mass was performed; histological analysis revealed inflammatory tissue. Surgical excision of the mass was performed; histopathological analysis revealed metastases from squamous carcinoma.

**Figure 1:**
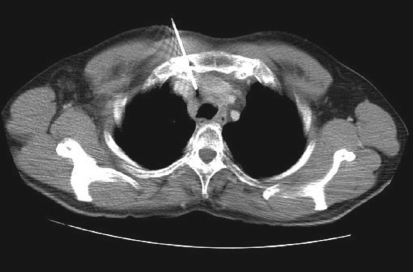
Trans-sternal approach for biopsy of pre-tracheal node in a treated case of renal cell carcinoma on follow up. PET-CT showed hypermetabolism in this node. Biopsy results revealed tuberculous lymphadenitis

Of the 80 diagnostic biopsies, 74 biopsy samples were definitive for neoplastic pathology. Six biopsy samples revealed no evidence of malignancy and were suggestive of non-neoplastic pathology. Out of these 6 non-neoplastic pathologies, 5 were tuberculous in origin and 1 was sarcoidosis which presented as mediastinal adenopathy. One of these patients was a treated case of renal cell carcinoma of right kidney. Follow-up PET-CT scan revealed hypermetabolism in the neck and mediastinal nodes. The mediastinal node was sampled under CT guidance; histopathologic diagnosis was tuberculous lymphadenitis [Figure 1]. All these patients responded to medical management, antitubercular therapy in case of tuberculous lymphadenitis and steroids in case of sarcoidosis.

Out of these 74 biopsies, 24 had surgical pathology confirmation. These patients were either primarily operated upon or treated initially with chemotherapy or radiotherapy and later underwent surgery to remove the residual tumor. The biopsy diagnoses in these cases were thymoma (n = 9), neurogenic tumor (n = 5), teratoma (n = 3), malignant round cell tumor (n = 4), neuroblastoma (n = 1), liposarcoma (n = 1) and pleomorphic sarcoma (n = 1). In all these patients, the biopsy diagnoses were confirmed after examination of the excision specimen.

In 51 biopsies, surgical confirmation was not possible. In 24 of these cases, biopsy diagnoses were concordant with the clinical, biochemical and radiological features of the tumor; the predicted response to therapy; or clinical behavior of tumor. The biopsy diagnoses in these cases were lymphoma (n = 20), yolk sac tumor (n = 1), residual primitive neuroectodermal tumor (n = 1), malignant germ cell tumor (n = 1), spindle cell sarcoma (n = 1).

Out of these 51 biopsies, 26 biopsy results were suggestive of metastases. The biopsy findings were corelated with histopathology of primary malignancy. The biopsy diagnoses in these cases were metastases from adenocarcinoma (n = 8), squamous carcinoma (n = 6), small cell carcinoma of lung (n = 5), non-small cell carcinoma of lung (n = 4), osteogenic sarcoma (n = 1), epithelioid sarcoma (n = 1) and pleomorphic sarcoma (n = 1).

There were no major complications that required any kind of intervention. Five minor complications such as small pneumothorax (n = 4) and hematoma (n = 1) were recorded. No active intervention was required in these cases. Patients were observed for hemodynamic stability and respiratory status for at least 3 h and then discharged.

## Discussion

In mediastinal lesions, early diagnosis, separation of malignant-benign lesions and early medical treatment are the main goals to decrease mortality. The possibility of severe complications such as respiratory or circulatory compression mandates fast and sometimes urgent diagnosis and treatment of mediastinal masses.

There are several methods for obtaining tissue samples for cytologic or histologic diagnosis of mediastinal lesions. These include percutaneous image-guided transthoracic needle biopsy; surgical techniques such as thoracoscopy, cervical mediastinoscopy and anterior mediastinotomy; and needle biopsy techniques include transbronchial needle biopsy and endoscopic ultrasound-guided fine needle aspiration biopsy.[9–14]

CT-guided percutaneous transthoracic needle biopsy has several advantages over other alternative biopsy techniques. Image guidance allows precise localization of target lesion and access to virtually all mediastinal compartments, including those that are inaccessible with other alternative methods, such as mediastinoscopy, transbronchial biopsy and endoscopic ultrasound-guided biopsy.

Percutaneous transthoracic biopsy is less invasive than mediastinoscopy and requires only local anesthesia. In comparison to other alternative methods, transthoracic biopsies are cost-effective because they shorten the period from admission to diagnosis, decrease the number of surgical procedures and shorten the time of hospital stay, which reduces overall treatment costs.[11] There are only few contraindications that preclude the use of percutaneous biopsies in every case, absolute contraindication being suspicion of hydatid cyst; whereas relative contraindications are bleeding diathesis, bullous emphysema, pulmonary hypertension and a high rank of vascular tumor.[11–13]

**Figure 2:**
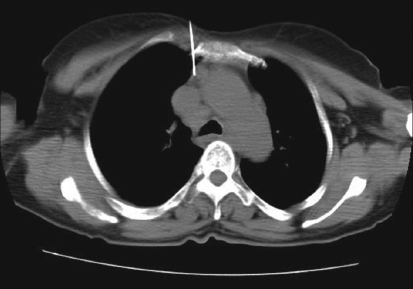
Para-sternal approach for biopsy of pre-vascular node. Needle advanced between sternum and internal mammary vessels. Histopathological analysis revealed metastases from squamous cell carcinoma

**Figure 3:**
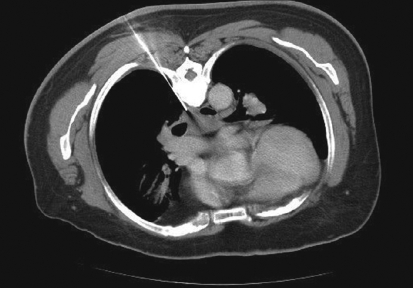
Para-vertebral approach for biopsy of subcarinal node. Histopathological analysis revealed tuberculous lymphadenitis

**Figure 4:**
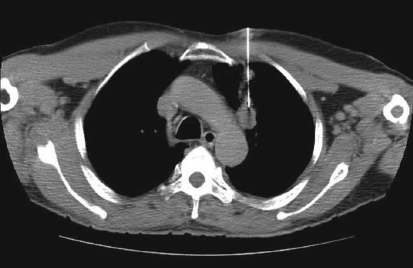
Transthoracic approach for biopsy of prevascular node. Biopsy results revealed metastases from adenocarcinoma

**Figure 5:**
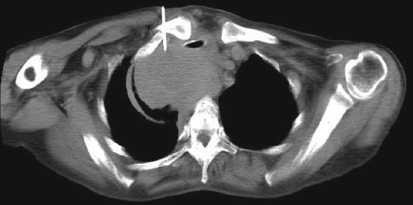
Suprasternal approach for biopsy of superior medaistinal mass. Histopathological analysis revealed germ cell tumour

Various approaches for image-guided transthoracic biopsies of mediastinal lesions have been advocated. These include extrapleural or direct mediastinal approaches such as parasternal, paravertebral, trans-sternal and subxiphoid approaches; and the transpulmonary approach[13,14] [Figures 1-5]. The best approach to biopsy of a mediastinal lesion depends on the location and size of target lesion and the presence of any comorbid conditions. In our series, approaches for targeting mediastinal lesions were parasternal (n = 27), paravertebral (n = 24), transpulmonary (n = 23), suprasternal (n = 5) and trans-sternal (n = 4).

In our series, repeat attempt to sample the mediastinal mass was made once. The second biopsy result in this patient was diagnostic of Hodgkin's disease. Out of 83 biopsies, 80 biopsy results were considered diagnostic (96.4%) in whom the results could be confirmed by surgical pathology, clinical follow-up examination or correlation with primary malignancy in cases of metastatic diseases. This is in concordance with other series that have dealt with percutaneous biopsy of mediastinal masses. In all these studies, the percutaneous biopsy results were shown to be correct in 75-90% of patients.[2,14–19]

Out of 83 biopsies, distribution of results were primary neoplasm (n = 49), secondary neoplastic process (n = 28) and non-neoplastic conditions (n = 6). In our series, diagnostic accuracy for primary mediastinal neoplasm was found to be 93.8%; and secondary neoplastic process, 96.5%. High rate of neoplastic pathologies in our study is due to our being a tertiary referral center for cancer. The diagnostic accuracy for non-neoplastic conditions was 100%. These results are in concordance with other studies.[16,20]

Lymphoma was the most common neoplastic pathology in our study group. Diagnostic accuracy of lymphoma in our series was 95.2%. There was no major complication; only five minor complications were recorded. No active intervention was required in these cases. Complication rate was 6.02%. Our results have shown percutaneous image-guided biopsy of mediastinal lesions to be accurate and safe.

## Conclusion

An overall diagnostic accuracy of 93-96% was achieved for percutaneous biopsy of mediastinal lesions in our series. Minor complications were seen in five patients (6.02%), and there were no major complications. Familiarity with cross-sectional imaging facilitates planning of safe access route for biopsy of mediastinal lesions. To conclude, percutaneous image-guided core biopsy of mediastinal lesions is safe, accurate and cost-effective.
